# Laparoscopic versus open surgery in obstructive colorectal cancer patients following stents placement: a comprehensive meta-analysis of cohort studies

**DOI:** 10.1007/s00464-024-10710-4

**Published:** 2024-03-05

**Authors:** Kerui Zeng, Faqiang Zhang, Hua Yang, Xiaoying Zha, Shixu Fang

**Affiliations:** 1 Department of Colorectal Anal Surgery, Zigong Fourth People’s Hospital, Zigong, Sichuan China; 2 Department of Wound Care Center, Department of Colorectal Anal Surgery, Zigong Fourth Hospital, Zigong, Sichuan China; 3 Department of Colorectal Anal Surgery, Zigong First People’s Hospital, Zigong, Sichuan China; 4 Department of General Surgery, Zigong Fourth People’s Hospital, No.282, Dangui Street, Ziliujing District, Zigong, 643000 Sichuan People’s Republic of China

**Keywords:** Colorectal cancer, Stent insertion, Laparoscopic surgery, Open surgery, Postoperative complications

## Abstract

**Background:**

Over the past decade, the use of stent placement as a bridge to surgery (BTS) has emerged as an alternative to emergency surgery for patients with (OCRC). However, the optimal surgical approach remains indeterminate. This study seeks to evaluate the safety and feasibility of a combined treatment modality involving stent placement and laparoscopic surgery for OCRC presenting with malignant obstruction.

**Methods:**

A comprehensive search of PubMed, Cochrane Library, EMBASE, Web of Science, and ClinicalTrials.gov was conducted until June 2023 to identify studies that compared laparoscopic to open surgery in patients with OCBC following stent insertion.

**Results:**

The meta-analysis incorporated 12 cohort studies, encompassing 933 patients. There was no statistically significant difference in the 30-day mortality rates between the two groups (relative risk [RR], 1.09; 95% confidence interval [CI] 0.26 to 4.48; *P* = 0.95). Compared to the laparoscopic approach group, the open approach group had a higher rate of overall postoperative complications (POCs) (RR 0.52; 95% CI 0.37 to 0.72, *P* < 0.0001). There was no significant variance in lymph node (LN) dissection number between the groups (mean differences [MD], 1.64; 95% CI − 1.51 to 4.78; *P* = 0.31). Notably, laparoscopic surgery resulted in less intraoperative blood loss (MD, − 25.84 ml; 95% CI − 52.16 to 0.49; *P* = 0.05) and a longer operation time (MD, 20.99 mins; 95% CI 2.31 to 39.44; *P* = 0.03). The laparoscopic approach was associated with a shorter length of hospital stay (LOS) (MD − 3.29 days; 95% CI − 5.27 to 1.31; *P* = 0.001). Conversely, the open approach group had a higher rate of postoperative surgical site infection (SSI) (RR 0.47; 95% CI 0.23 to 0.96, *P* = 0.04). Although the number of included studies was insufficient to conduct a meta-analysis, several of them imply that laparoscopic surgery may yield more favorable outcomes in terms of the 3-year overall survival rate (OS), 3-year disease-free survival rate (DFS), 5-year OS, and 5-year DFS when compared to open surgery. It is worth noting that these differences lack statistical significance.

**Conclusion:**

In patients with OCRC subjected to stent insertion, laparoscopic surgery arguably presents a modest superiority over open surgery by diminishing the overall postoperative risk and potentially reducing the LOS.

**Supplementary Information:**

The online version contains supplementary material available at 10.1007/s00464-024-10710-4.

Colorectal cancer (CRC), the third most frequently diagnosed and deadly cancer globally, yields an estimated 1.85 million new cases annually [1, 2]. The primary objective is high-quality oncological resection [3]; however, an estimated 8% to 29% of CRC patients present with acute colorectal obstruction (ACO) [4]. Often, these patients are older and frail and their clinical condition deteriorated due to insufficient oral intake [5]. The management of this severe condition remains a contentious issue [6].

Historically, patients with OCRC have undergone emergency open surgery to restore luminal patency [7]. Emergency surgery for OCRC patients, while effective in relieving obstruction either curatively or palliatively with a permanent stoma, is associated with high mortality and morbidity rates [8, 9]. In the past decade, self-expanding metal stents (SEMS) have proven to be safe, easy, and effective as bridge to surgery (BTS) and palliative treatment for CRC, with an acceptable complication rate [10]. It allows for the conversion of an emergency surgery to an elective one, reduces stoma creation rate, overall postoperative complications, and enables the use of laparoscopic techniques by improving the surgical field [11]. The European Society of Gastrointestinal Endoscopy (ESGE) guideline [6] recommend stenting as a BTS in patients with potentially curable left-sided obstructing colon cancer (LSOCC). The American Gastroenterological Association (AGA) clinical practice [12] suggests inserting SEMS as a BTS for patients with proximal (or right-sided) malignant obstructions who are potential candidates for resection, thus facilitating elective rather than emergency surgery. A propensity score-matched analysis of oncological outcomes [13], as well as several meta-analyses [14, 15], has confirmed the feasibility, oncological safety, and benefits of SEMS implantation, which in certain cases can serve as a bridge to later elective surgery. Studies [16–18] have also demonstrated that a laparoscopic approach is feasible for patients with OCRC due to CRC following stent placement.

However, laparoscopic surgery presents unique challenges for OCRC patients, such as the introduction of trocars into a distended abdomen, potential intestinal injury, limited instrument mobility, and increased difficulty due to large, locally advanced tumors causing luminal obstruction. In contrast, laparotomy, while straightforward and versatile, is more traumatic and imposes a slower recovery post-operation. Comparative studies on the efficacy of laparoscopic surgery and laparotomy following stent placement are limited and a clear consensus on the surgical approach for OCRC patients post-SEMS implantation is lacking. Questions remain on the optimal time interval, technical difficulty, and other issues from stent implantation to determining the operation. Thus, this study aims to compare the safety and efficacy of elective laparoscopic surgery and open surgical approaches following stent insertion for OCRC patients.

## Method

The development of the inclusion criteria adhered to the guidelines set forth by the Cochrane Collaboration [19] and the recommendations from the Preferred Reporting Items for Systematic Reviews and Meta-Analyses (PRISMA) [20]. The methodology of the study is documented in a protocol, which is registered and accessible at http://www.crd.york.ac.uk/prospero/ (registration number: CRD42022301124).

### Search strategy

A comprehensive literature search was conducted using PubMed, Cochrane Library, EMBASE, Web of Science, and ClinicalTrials.gov with the key terms ‘colorectal neoplasms,’ ‘intestinal obstruction,’ ‘stents,’ ‘laparoscopy,’ ‘minimal access surgical,’ and ‘elective surgery,’ without any restrictions on language or date. The search strategy is provided in Online Appendix 1. The initial search was performed in March 2022 and an email alert was set up in databases and journals to receive notifications for any new publications. However, no new relevant studies were identified, suggesting that the search period was from March 2022 to the end of February 2023. Each article was reviewed and analyzed by at least two members of the research team (KR.Z. and SX.F.) using an unblinded standardized approach for eligibility assessment. In cases where multiple publications reported findings for the same patients, the most recent or most comprehensive study was selected. Any conflicting studies were resolved through discussions among all the authors.

### Literature inclusion

#### Inclusion criteria

Studies satisfying the subsequent criteria were deemed eligible for inclusion: (1) Patients who have undergone surgical intervention following stent insertion as a BTS for malignant obstruction induced by CRC; (2) The study compared the outcomes of stent insertion combined with laparoscopic surgery (experimental group) and stent insertion combined with laparotomy (control group); (3) The study reported a minimum of one outcome of interest; and (4) The study was a prospective study, retrospective study, or a Randomized Controlled Trial (RCT) published in either English or Chinese.

#### Exclusion criteria

Studies were excluded if they met the following criteria: (1) The administration of any adjuvant treatments, such as chemotherapy, between SEMS insertion and surgery; (2) Incomplete data; (3) Instances of repeat publications or studies with duplicate data, retaining only the highest quality representation; and (4) Studies that do not permit the execution of a meta-analysis.

### Outcomes of interest and definitions

Primary outcomes included the following: (1) 30-day mortality rate; (2) overall postoperative complications (POCs) rates; (3) 3- and 5-year DFS rates; and (4) 3- and 5-year OS rates. The term ‘overall POCs’ encompasses any diagnosed adverse events associated with the surgery prior to hospital discharge.

Secondary outcomes included lymph node (LN) dissection and a range of perioperative outcomes, such as operation duration, intraoperative blood loss, length of hospital stay (LOS), time to first flatus, and any postprocedural adverse events prior to hospital discharge, including postoperative ileus (POI), surgical site infection (SSI), anastomotic leakage (AL), postoperative pulmonary infection, and postoperative wound dehiscence.

### Data extraction

Two researchers, KR.Z. and SX.F., independently reviewed the articles. Any disagreements regarding the inclusion of certain studies were resolved through discussions involving all authors. The first step involved identifying and excluding any duplicate articles. Subsequently, these two researchers analyzed the titles and abstracts of the articles, eliminating those that were irrelevant. The full texts of the remaining articles were then scrutinized for potential inclusion. KR.Z. conducted the data extraction, which was later reviewed and confirmed by SX.F. Any discrepancies were resolved through discussion among all authors. Where available, the reviewers independently gathered four types of data. The first type included basic information about the studies, such as titles, authors, publication year, and country of origin. The second type involved baseline characteristics, including study design, tumor location, sample size, patient age and sex, American Society of Anesthesiologists (ASA) classification, time from SEMS insertion to surgery, and median follow-up period. The third type of data related to outcomes of interest, encompassing perioperative outcomes, histopathological outcomes, and postoperative complications. The fourth type included key elements for assessing bias risk.

### Quality evaluation

Two reviewers, KR.Z. and FQ.Z., independently assessed the risk of bias in individual studies using the Newcastle–Ottawa Quality Assessment Scale (NOS) [21]. The NOS evaluation is based on three specific domains: selection, comparability, and outcome, with scores ranging from zero to nine. In our study, scores equal to or greater than seven were deemed high quality, while scores lower than seven were considered low quality. RCTs were evaluated according to the Cochrane Collaboration guidelines [19] to identify potential bias. These potential biases included random sequence generation, allocation concealment, blinding of outcome assessment, blinding of participants, selective reporting, assessment of incomplete data outcome, and other potential sources of bias.

### Statistical analysis

Data analysis was conducted using RevMan software (Cochrane Review Manager, Version 5.4.1) and STATA version 16.0 (StataCorp LP, College Station, TX). Relative risks (RR) and 95% confidence intervals (CIs) for dichotomous variables were estimated via the Mantel–Haenszel method. For continuous outcome data, mean differences (MDs) and 95% CIs were calculated using inverse variance weighting. In cases where means or standard deviations (SDs) were not provided, estimations were made from the reported medians, ranges, and sample size as outlined by Hozo et al. [22]. Forest plots were then generated using RevMan, with a *P* value of less than 0.05 on 2-tailed testing indicating a statistically significant difference. If both the exposure and control groups in a study had a value of zero, STATA was utilized to generate a forest plot. A 0.5 continuity correction transformation was implemented to account for zero events. To assess heterogeneity, the Q test based on the *χ*^2^ statistics and *I*^2^ statistics were used. Significant between-study heterogeneity was determined when *P* < 0.1 and *I*^2^ > 50% among the studies. Anticipating between-study heterogeneity, the pooled estimates were analyzed using the random-effects model and the Der Simonian and Laird method based on the moment estimator. Subgroup analysis, meta-regression, and sensitivity analysis were employed to analyze significant heterogeneity, or it was merely stated. Sensitivity analysis was executed by sequentially eliminating individual studies. Publication bias was initially assessed by visually inspecting for the presence of funnel plot asymmetry, and the Egger test was used to evaluate the presence of asymmetry, with *P* < 0.05 deemed significant.

## Results

### Study selection

**Fig. 1:**
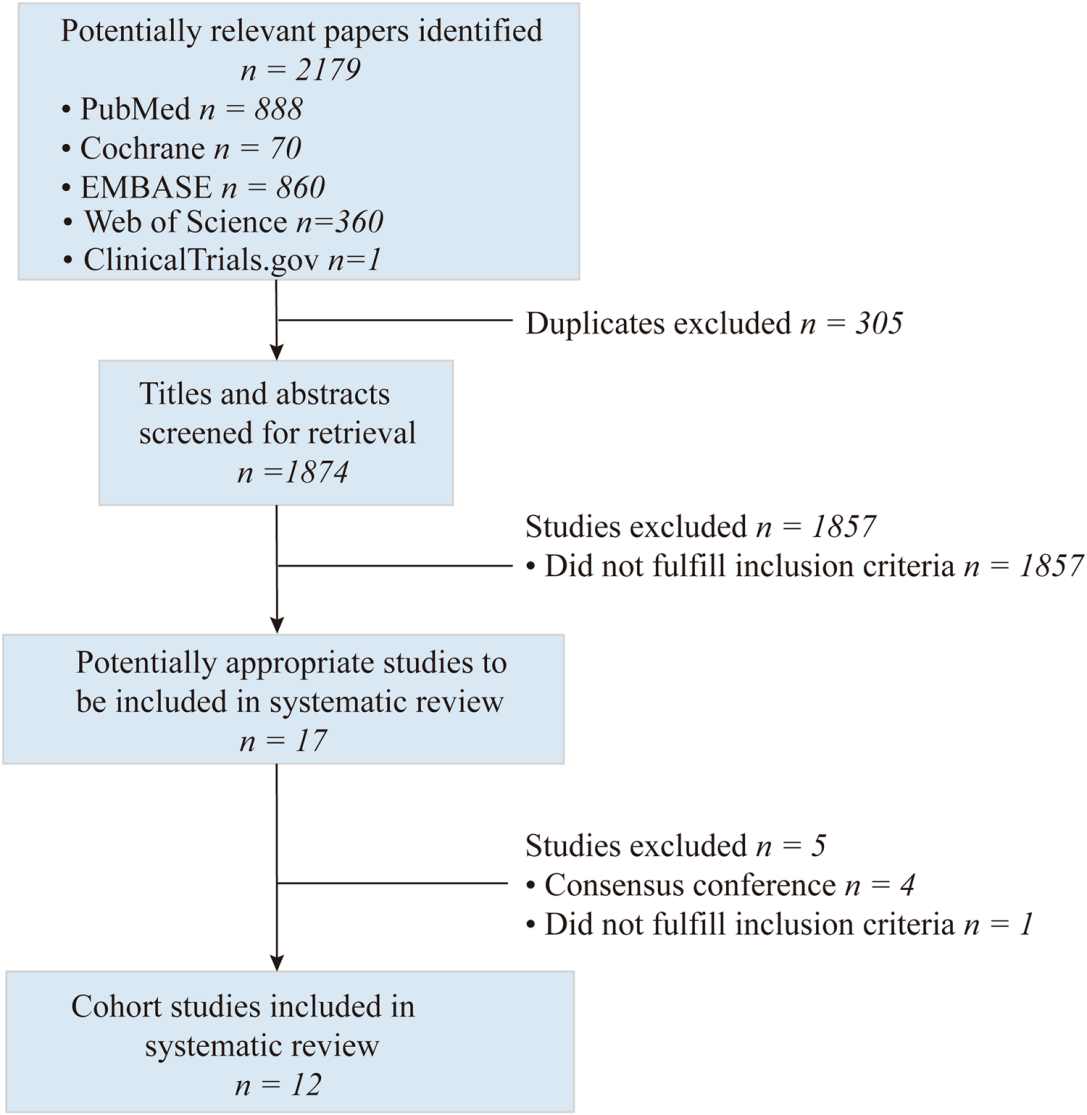
Flowchart of study selection.

Figure 1 illustrates the detailed procedures of the systematic literature selection. Initially, a total of 2179 articles were identified via a systematic review of the literature. After eliminating 305 duplicate papers from the initial search, the number was reduced to 1874. Of these, 1857 studies were excluded after examining titles and abstracts due to non-compliance with the inclusion criteria. Notably, four consensus conference studies and one clinical trial [23] were among the excluded. The clinical trial’s patient data did not meet the inclusion criteria. A thorough review of the full text of the remaining 17 potentially suitable studies led to the inclusion of 12 cohort studies [16, 24–34] in the quantitative analysis. Of these, 11 were retrospective studies, while one was a prospective study [34].

### Characteristics of included studies

The studies under consideration encompassed a total of 933 patients who underwent either laparoscopic or open surgical procedures. Of these, 463 patients were part of the laparoscopic group, while the open group consisted of 470 patients. A radical effect was achieved in the types of surgeries reported in four studies [26, 28, 31, 33]. Another set of four studies [24, 25, 32, 34] incorporated both radical and non-radical surgeries. However, the surgical types, whether curative or otherwise, were not specified in the remaining four studies [16, 27, 29, 30]. A total of 14 outcome indexes were compared, which included 30-day mortality, overall POCs, 3-year or 5-year DFS rates, 3-year or 5-year OS rates, LN dissection, operation time, intraoperative blood loss, LOS, POI, SSI, AL, postoperative pulmonary infection, postoperative wound dehiscence, and time to first flatus. The primary characteristics of the included studies are detailed in Table 1.

**Table 1:** The characteristic of the included studies

Author	Country	Design	Tumor site	Types of surgeries	Laparoscopic approach					Open approach					Outcomes	Median follow-up (mean)
					Sample size	Age (year) (mean)	Male (%)	ASA class (I/II/III/IV)	Times from sems to surgery (mean)	Sample size	Age (year) (mean)	Male (%)	ASA class (I/II/III/IV)	Times from sems to surgery (mean)		
Kim et al. (2020)	Korea	R, multicenter	Left-side colon; upper rectum	NA	97	63.4 ± 12.7	53.60%	32/56/9/-	6–10 days (8 days)	82	64.1 ± 13.6	65.90%	21/52/9/–	5–12 days (6 days)	①③⑤⑥⑦⑧	47.3 months for LAP; 52.5 months for OPEN
Tajima et al. (2020)	Japan	R, single center	Right-side colon; left-side colon; rectum	Radical and non-radical	54	63.5 (30–82)	61.10%	21/33/- (I/II + III/IV)	0–62 days (13 days)	21	68.0 (50–86)	71.40%	4/17/- (I/II + III/IV)	0–62days (13 days)	④⑤⑥⑦⑧⑨⑩⑪	28 months for LAP; 25.0 months for OPEN
Yang et al. (2019)	Korea	R, single center	Right-side colon; left-side colon; rectosigmoid colon	Radical	105	66.5 ± 12.3	56.20%	71/34 (I + II/III + IV)	6–12 days (8.0 days)	77	63.4 ± 12.4	62.30%	56/21 (I + II/III + IV)	6–14 days (8.0 days)	②⑥⑦⑧⑨⑩⑪⑫⑬⑭	60.4 months
Bae et al. (2019)	Korea	R, multicenter	Right-side colon; left-side colon; rectosigmoid colon	Radical	44	69 (45–88)	81.80%	17/20/4/-	2–55 days (11 days)	50	66 (40–83)	52.00%	24/23/3/–	0–101 days (12 days)	①②③⑤⑥⑧⑨⑩⑪⑫⑬	47 months for LAP; 48 months for OPEN
Lu et al. (2017)	China	R, single center	Right-side colon; left-side colon; rectum	Radical	34	64.1 ± 11.2	73.50%	NA	7–20 days (10 days)	18	65.7 ± 12.5	66.70%	NA	7–20 days (10 days)	②⑤⑥⑦⑧⑨⑩⑫⑭	NA
Li et al. (2016)	China	R, single center	Right-side colon; left-side colon; rectum	Radical	41	NA	NA	NA	2–59 days (12 days)	79	NA	NA	NA	2–59 days (12 days)	①②⑤⑥⑦⑧⑨⑩⑪⑫⑭	29 months
Enomoto et al. (2016)	Japan	R, single center	Right-side colon; left-side colon; rectum	NA	26	65	57.69%	NA	9 days	32	75	65.62%	NA	10 days	①②⑥⑦⑧⑨⑩⑪	NA
Zhou et al. (2013)	China	R, single center	Descending colon; sigmoid colon; rectum	NA	14	57.7 ± 9.6	71.42%	NA	13.9 ± 13.2 days	58	60.2 ± 12.8	62.06%	NA	10.6 ± 13.3 days	②③④⑥⑦⑧⑨⑩⑪⑬⑭	28.2 months for LAP 28.9 months for OPEN
Law et al. (2013)	China	P, single center	Right-side colon; left-side colon; rectum	Radical and non-radical	18	70.5	72.22%	NA	4–110 days (11 days)	18	73.5	83.33%	NA	4–10 days (11 days)	①②	20 months
Chung et al. (2008)	Korea	R, single center	Descending colon; sigmoid colon; upper rectum	Radical and non-radical	17	69 (33–80)	47.05%	3/13/1/0	2–11 days (7 days)	8	61 (42–80)	37.50%	2/6/0/0	2–151 days (5 days)	②⑤⑥⑦⑧⑨	NA
Matsushima et al. (2017)	Japan	R, single center	Right-side colon; left-side colon; rectosigmoid colon	Radical and non-radical	7	NA	NA	NA	9–20 days (14 days)	11	NA	NA	NA	9–20 days (14 days)	②⑤⑥⑦⑨⑩⑪⑫	NA
Stipa et al. (2008)	Italy	R, single center	Transverse colon; left-side colon; rectosigmoid colon	NA	6	65.8 ± 5.3	83.33%	NA	6–18 days (13.8 days)	16	72.0 ± 11.5	50.00%	NA	1–21 days (8.9 days)	①②⑫	28 months

*NA* not provided; *ASA* American Society of Anesthesiologists; *LAP* Laparoscopic approach; *OPEN* Open approach; *R* retrospective; *P* prospective; *POCs* postoperative complications; *DFS* disease-free survival; *OS* overall survival; *LN* lymph node; *LOS* length of hospital stay; *POI* postoperative ileus; *SSI* surgical site infection; *AL* anastomotic leakage

Outcome indicators: ① the 30-day mortality rate; ② overall POCs rate; ③ 3-year or 5-year DFS rate; ④ 3-year or 5-year OS rates; ⑤ LN dissection; ⑥ the operation time; ⑦ the intraoperative blood loss; ⑧ LOS; ⑨ POI; ⑩ SSI; ⑪ AL; ⑫ postoperative pulmonary infection, ⑬ postoperative wound dehiscence; and ⑭ time to first flatus

### Risk of bias of the studies

The quality of the cohort studies was evaluated based on the NOS scores, which are detailed in Table 2. Quality assessments conducted on all the papers yielded scores ranging from 5 to 8. Nine studies [16, 26–29, 31–34] were deemed high quality (with a quality score of 7 or above), while the remaining three studies [24, 25, 30] were classified as low quality (with a quality score below 7).

**Table 2:** The associated risk of bias according to the NOS scores

Study	Selection				Comparability	Outcome			
	Representativeness of the exposed cohort	Selection of the non-exposed cohort	Ascertainment of exposure	Demonstration that outcome of interest was not present at start of study	Comparability of cohorts on the basis of the design or analysis	Assessment of outcome	Was follow-up long enough for outcomes to occur	Adequacy of follow-up of cohorts	All
Kim (2020)	1	1	1	1	1	1	1	0	7
Tajima (2020)	1	1	1	1	2	1	1	0	8
Yang (2019)	1	1	1	1	2	1	1	0	8
Bae (2019)	1	1	1	1	1	1	1	0	7
Lu (2017)	1	1	1	1	2	1	0	0	7
Matsushima (2017)	1	1	1	1	0	1	0	0	5
Li (2016)	1	1	1	1	0	1	1	1	7
Enomoto (2016)	1	1	1	1	0	1	0	0	5
Zhou (2013)	1	1	1	1	2	1	1	0	8
Law (2013)	1	1	1	1	1	1	1	0	7
Chung (2008)	1	1	1	1	0	1	0	0	5
Stipa (2008)	1	1	1	1	0	1	1	1	7

*NOS* Newcastle–Ottawa quality assessment Scale

### Primary outcomes

**Fig. 2:**
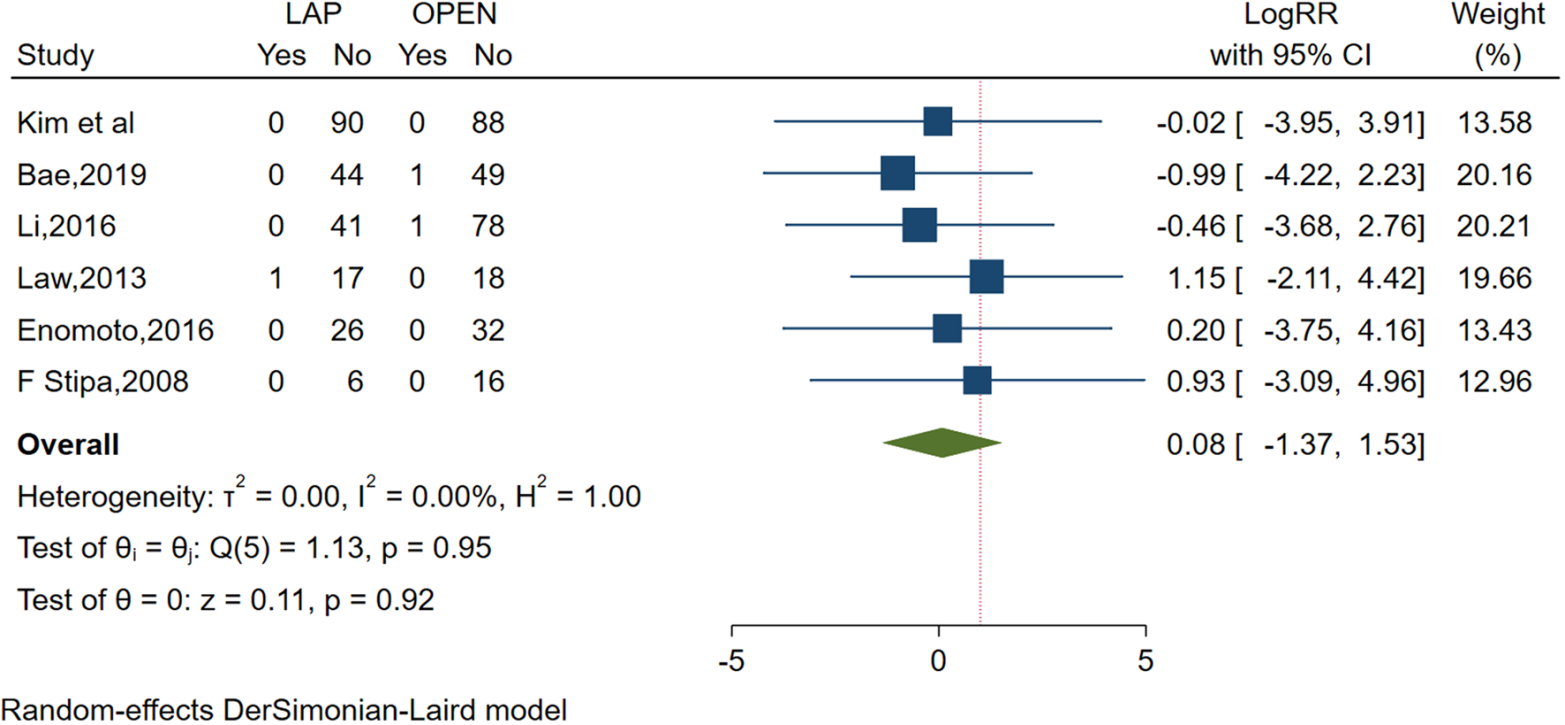
Frost plot for 30-day mortality rates. *LAP* laparoscopic approach; *OPEN* open approach

**Fig. 3:**
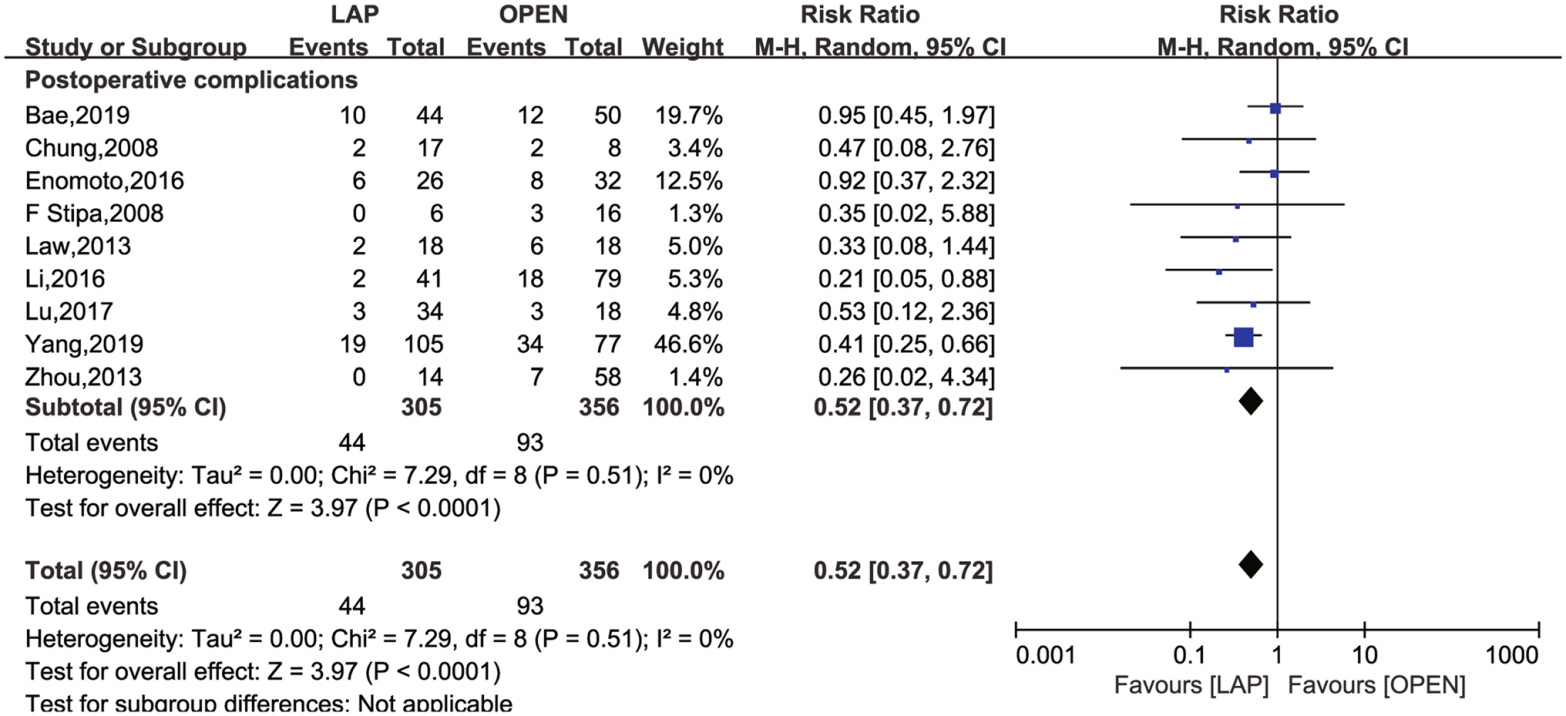
Frost plot for overall POCs rates. *POCs* postoperative complications; *LAP* laparoscopic approach; *OPEN* open approach

This meta-analysis examined the 30-day mortality rates (Fig. 2) and overall POCs rates (Fig. 3), along with 3-year and 5-year DFS and OS rates as the primary outcomes.

Six studies [26, 27, 29–31, 34] evaluated the 30-day mortality rates, revealing no significant differences (RR 95% CI 1.09; 0.26 to 4.48; *P* = 0.95), with no observed between-study heterogeneity (*I*^2^ = 0, P = 0.91). The rates of overall POCs were reported in nine studies [16, 25–28, 30, 31, 33, 34]. In comparison to the laparoscopic group, the open approach group exhibited a higher rate of overall POCs (RR 0.52; 95% CI 0.37 to 0.72, *P* < 0.0001), with no apparent between-study heterogeneity (*I*^2^ = 0, *P* = 0.51).

The study by reference [26] underscores a 5-year DFS rate of 61.5% within the laparoscopic group, in contrast to 55.8% in the open surgery cohort. However, these findings lack statistical significance (HR 0.982, 95% CI 0.522 to 1.847; *P* = 0.995). Despite the absence of statistical significance, the same study [26] reports a higher 5-year OS rate of 71.7% for the laparoscopic group, compared to the 67.1% rate in the open surgery group (HR 1.028, 95% CI 0.491 to 2.15; *P* = 0.942). Another study [32] documented both 5-year DFS and OS rates. Despite the laparoscopic approach group exhibiting higher 5-year DFS rates and 5-year OS rates, no significant differences were observed in either the 5-year DFS or OS rates when comparing the laparoscopic approach group with the open approach group (5-year DFS rates were 68.9% vs. 57.1%, *P* = 0.233; 5-year OS rates were 81.65% vs. 71.0%, *P* = 0.206).

Tajima’s investigation [32] revealed a 3-year OS rate of 66.4% for the laparoscopic approach group, slightly lower than the 67.5% rate observed in the open approach group, although the difference lacked statistical significance (*P* = 0.56). In the same study [35], a 3-year DFS rate of 82.2% was observed for the laparoscopic group, in contrast to 62.5% for the open group. Despite the laparoscopic group exhibiting a higher 3-year DFS rate, no statistical significance was found between the two groups (*P* = 0.11). Another investigation [16] disclosed no significant disparities between the laparoscopic and open approach groups in terms of 3-year DFS rates (71% vs. 70%, *P* = 0.731) or 3-year OS rates (83% vs. 70%, *P* = 0.915).

### Secondary outcomes

**Fig. 4:**
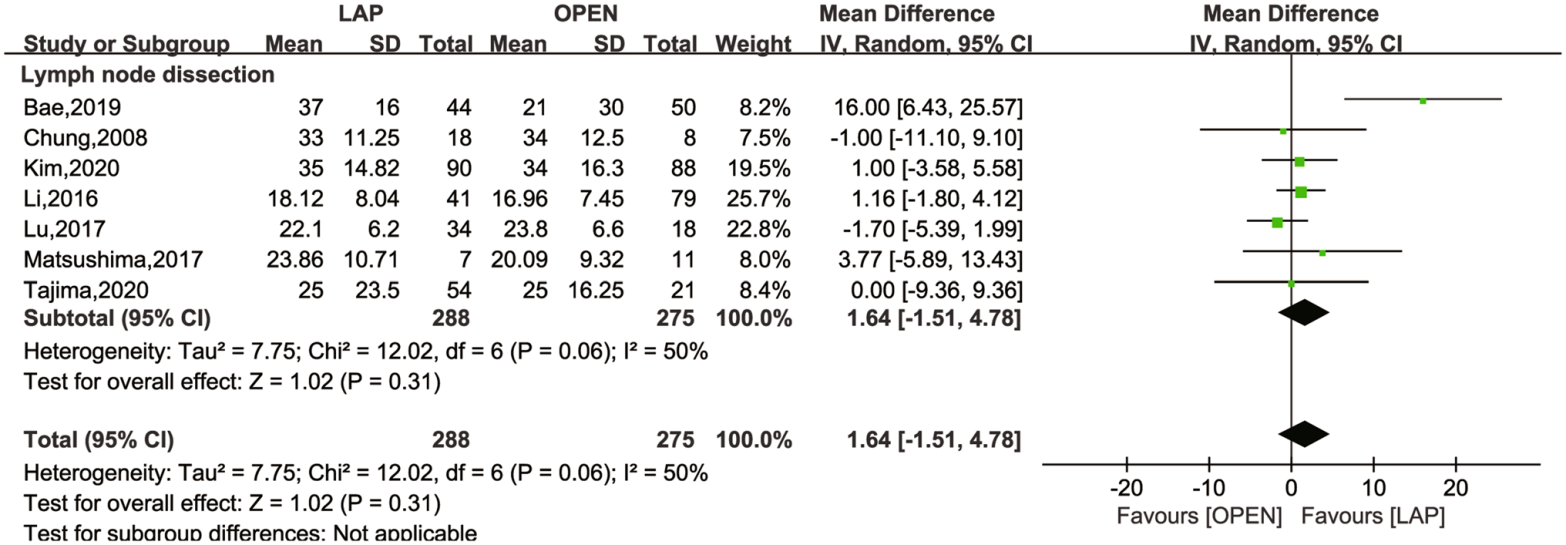
Frost plot for LN dissection. *LAP* laparoscopic approach; *OPEN* open approach

**Fig. 5:**
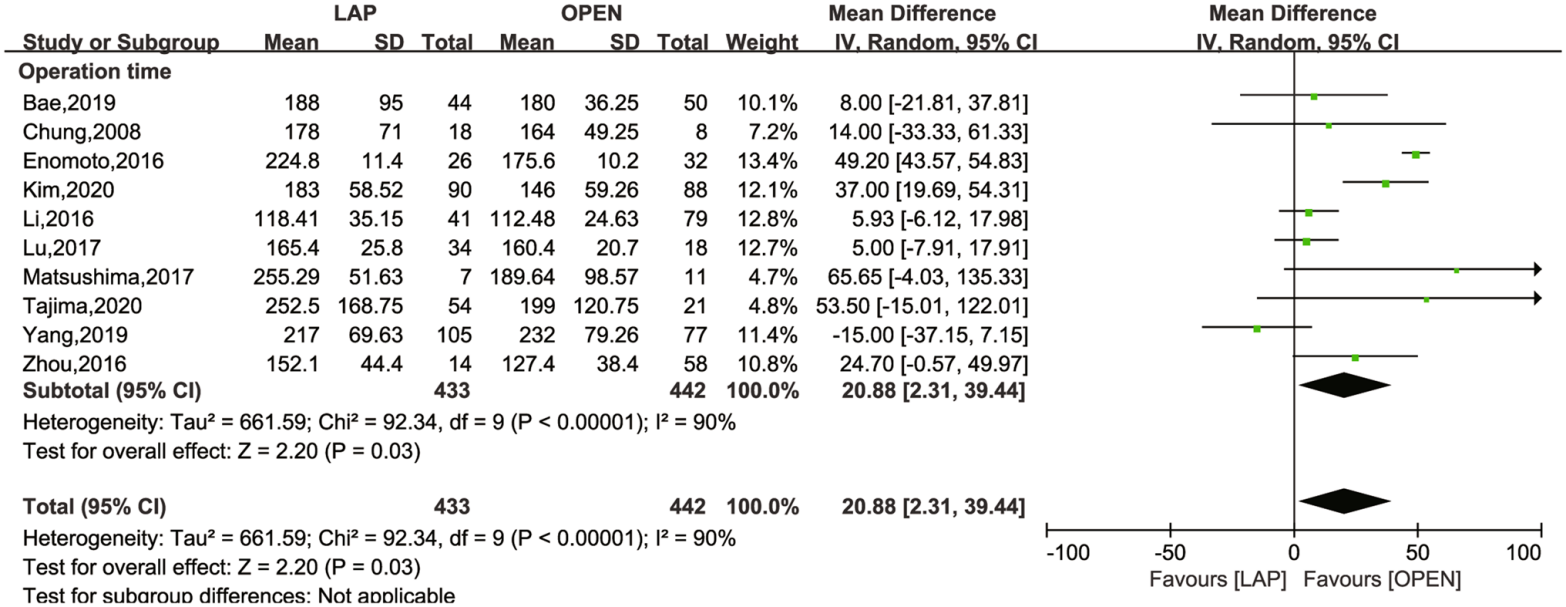
Frost plot for operation time. *LAP* laparoscopic approach; *OPEN* open approach

**Fig. 6:**
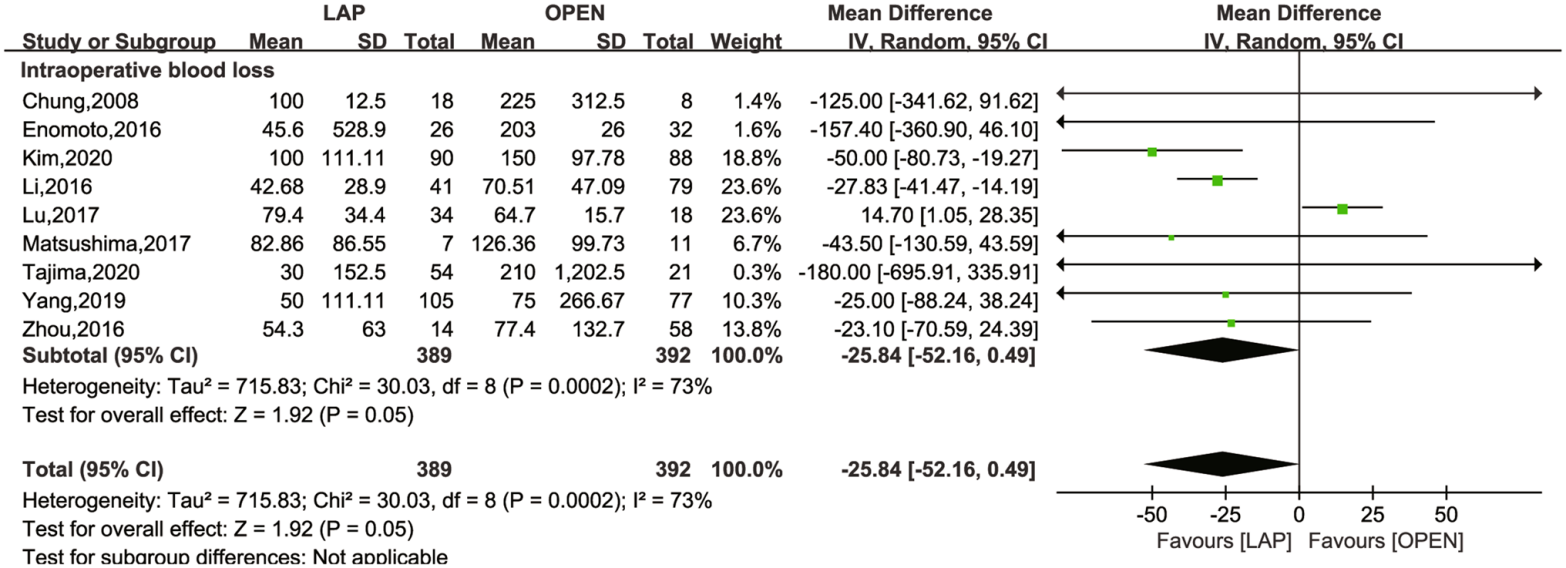
Frost plot for intraoperative blood loss. *LAP* laparoscopic approach; *OPEN* open approach

**Fig. 7:**
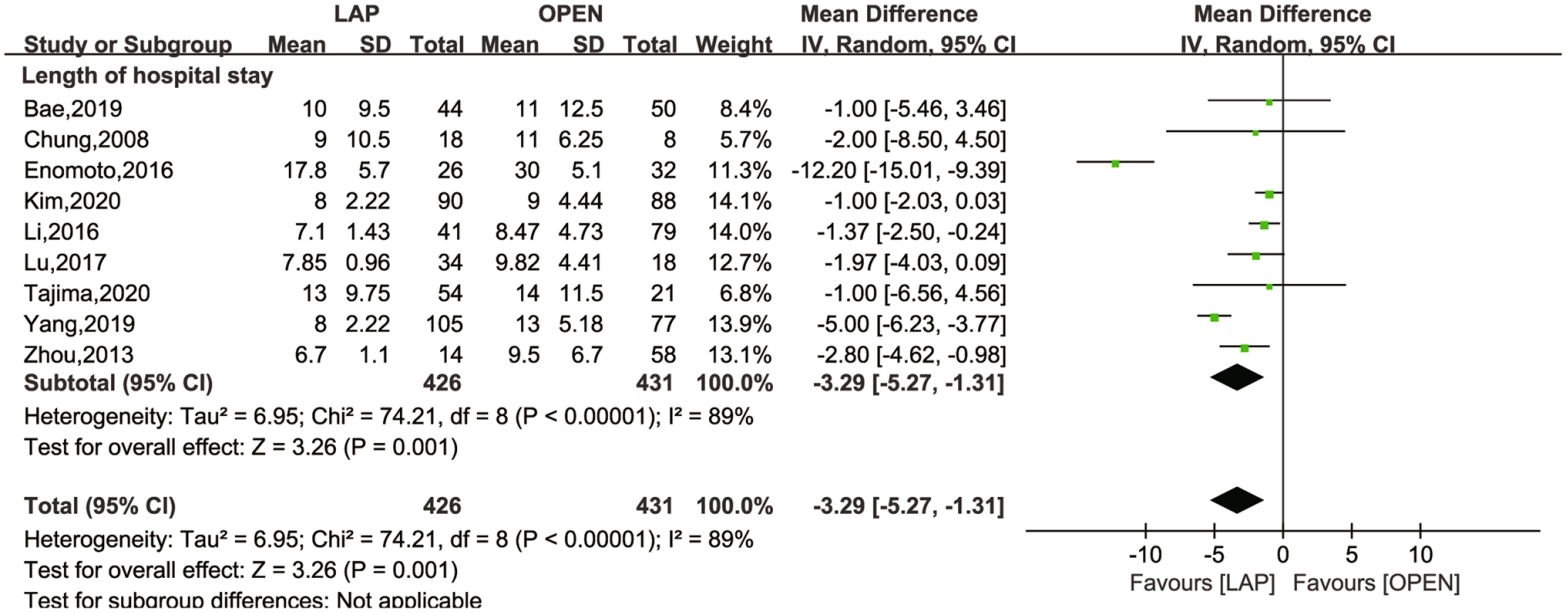
Frost plot for LOS. LOS: length of hospital stay. *LAP* laparoscopic approach; *OPEN* open approach

**Fig. 8:**
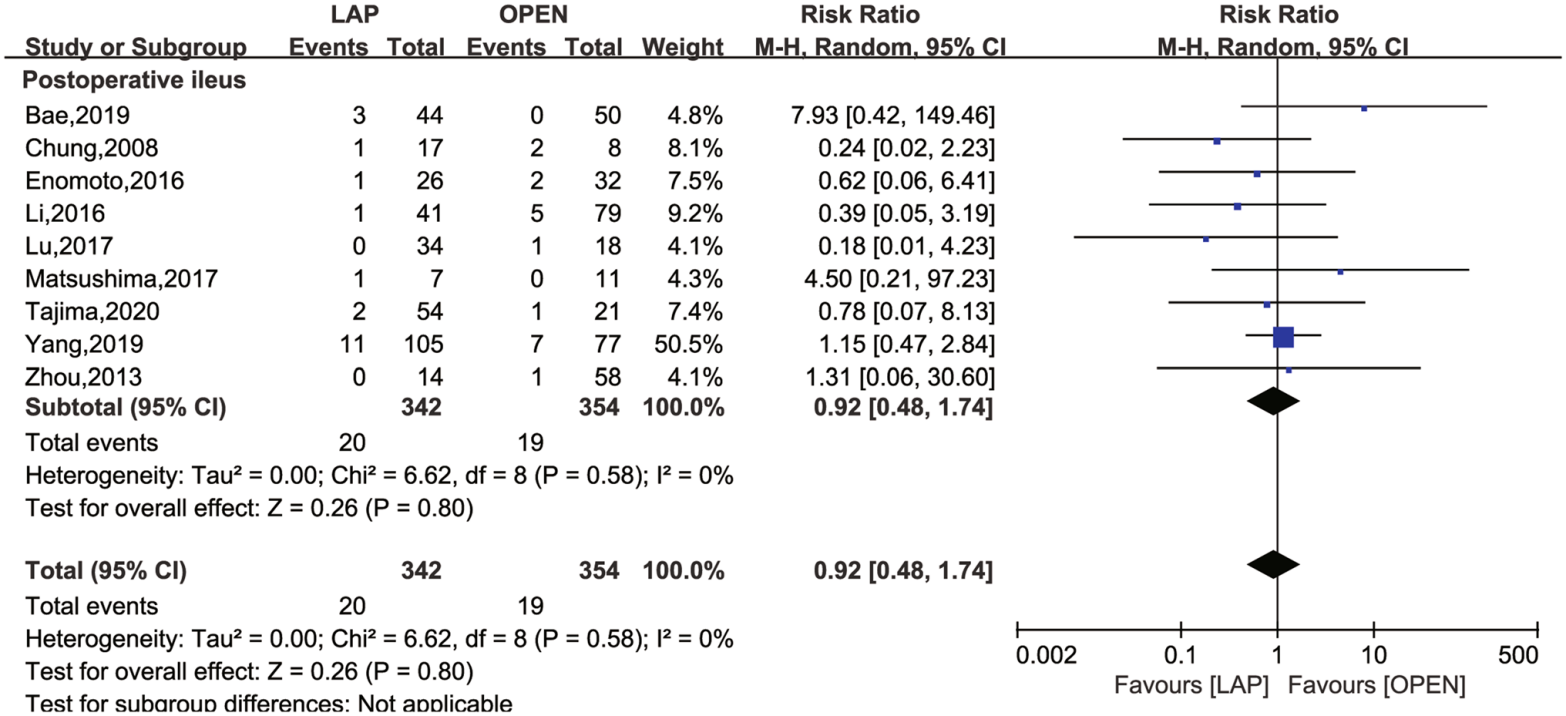
Frost plot for POI. *LAP* laparoscopic approach; *OPEN* open approach; *POI* postoperative ileus

**Fig. 9:**
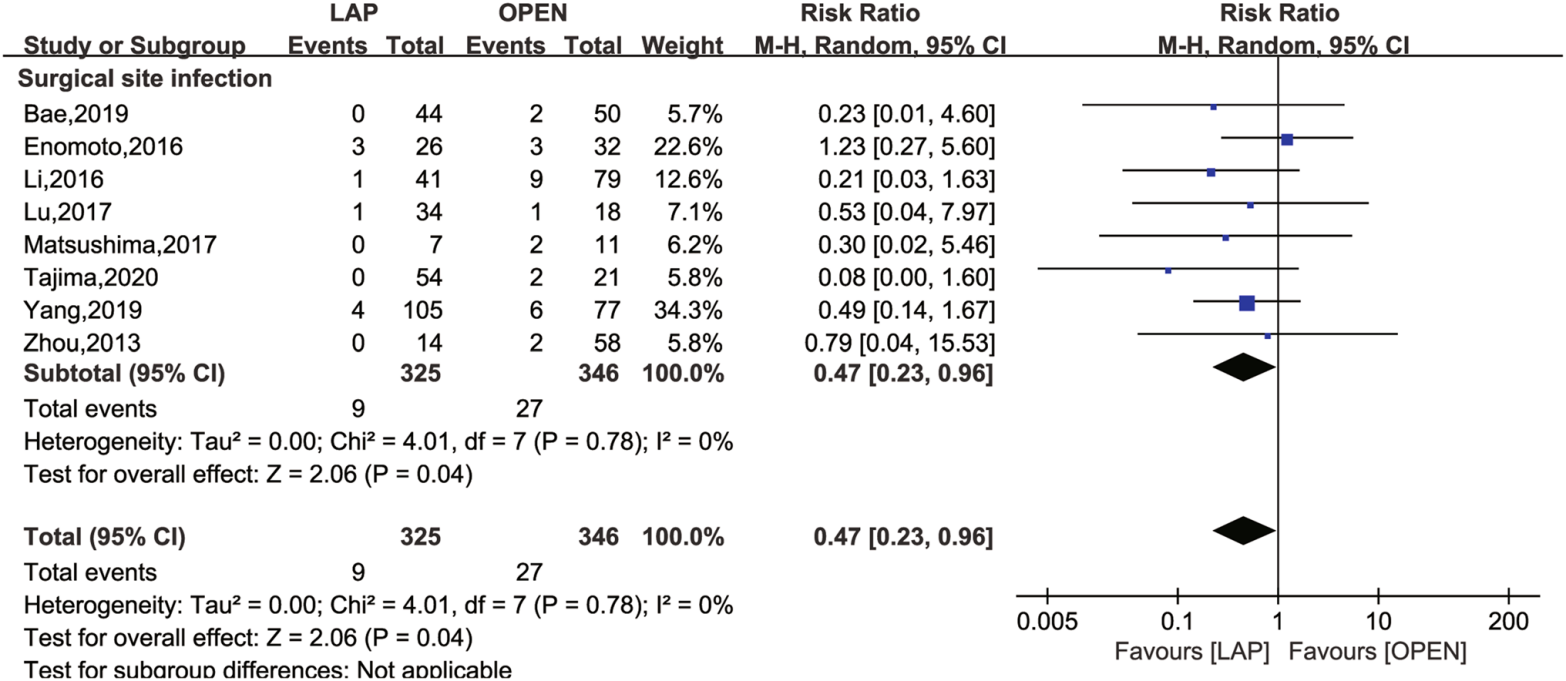
Frost plot for postoperative SSI. *SSI* surgical site infection; *LAP* laparoscopic approach; *OPEN* open approach

**Fig. 10:**
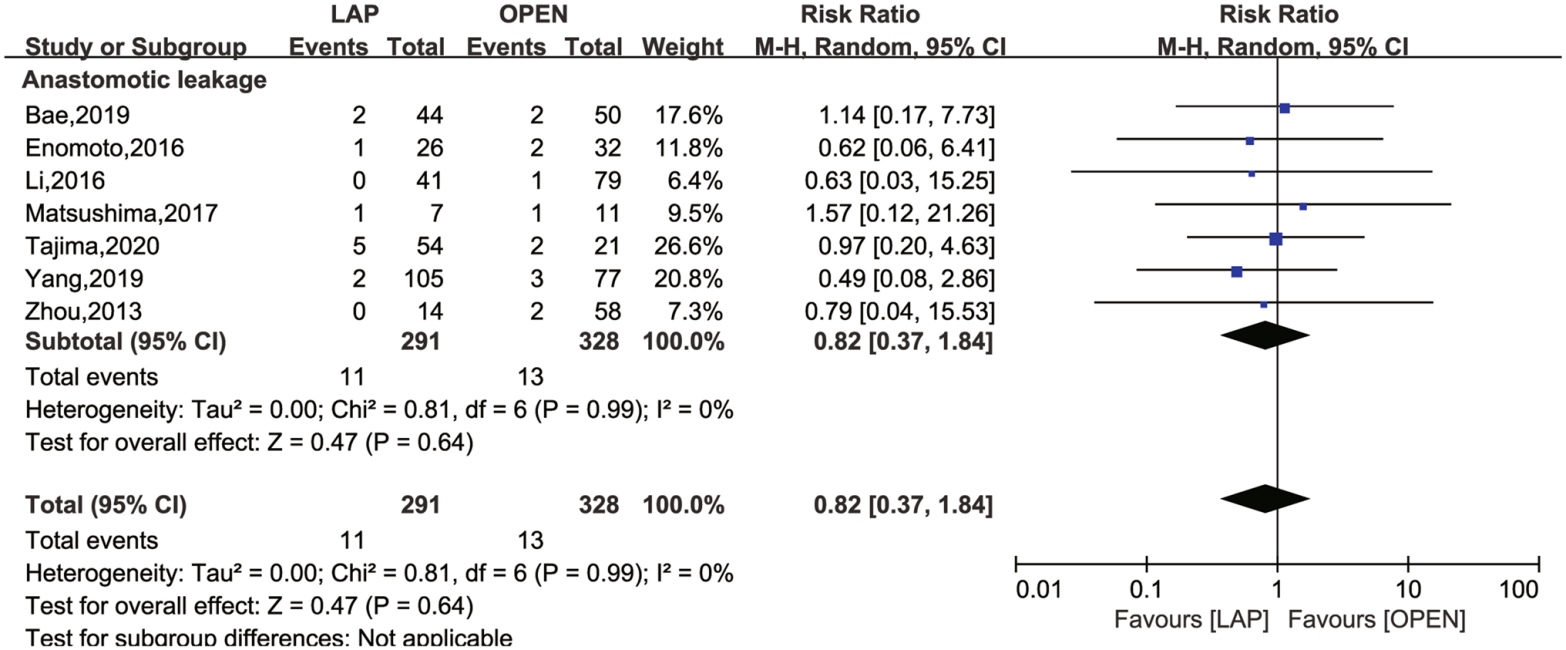
Frost plot for AL. *AL* anastomotic leakage, *LAP* laparoscopic approach; *OPEN* open approach

The secondary outcomes investigated comprised LN dissection (Fig. 4), operation time (Fig. 5), intraoperative blood loss (Fig. 6), LOS (Fig. 7), POI (Fig. 8), SSI (Fig. 9), and AL (Fig. 10).

Seven studies [24–26, 29, 31–33] reported on LN dissection, demonstrating a moderate level of heterogeneity (*I*^2^ = 50%, *P* = 0.06). The MD was 1.64 (95% CI − 1.51 to 4.78; *P* = 0.31), suggesting no significant difference between the groups in terms of LN dissection number. A sensitivity analysis, conducted by sequentially eliminating individual studies, confirmed the stability of the meta-analysis result, with no significant change observed pre- and post-elimination.

Operation time was documented in ten studies [16, 24–26, 28–33]. Despite a high degree of heterogeneity (*I*^2^ = 90% and *P* < 0.0001), a significant difference was noted between the laparoscopic approach and the open group (MD = 20.99 mins, 95% CI 2.31 to 39.44; *P* = 0.03). A funnel plot inspection showed no evidence of asymmetry, corroborated by an Egger test for funnel plot asymmetry resulting in a *P*-value of 0.4956, thus indicating no potential publication bias.

Intraoperative blood loss was reported in nine studies [16, 24, 25, 28–33] revealing a significant difference in favor of the laparoscopic approach (MD = − 25.84 ml, 95% CI − 52.16 to 0.49; *P* = 0.05), with considerable heterogeneity (*I*^2^ = 73% and *P* = 0.0002).

The six studies [25, 26, 29–31, 33] reported on LOS, exhibiting a high degree of heterogeneity (*I*^2^ = 89%, *P* < 0.00001). The overall effect test revealed a MD of -3.29 days, which was statistically significant (95% CI − 5.27 to 1.31; *P* = 0.001).

POI was assessed in nine studies [24–26, 28, 30–33], and no statistically significant risk difference between the two groups was found (RR 0.92; 95% CI 0.48 to 1.74; *P* = 0.8), in the absence of between-study heterogeneity (*I*^2^ = 0, *P* = 0.58).

SSI was reported in eight studies [16, 24, 26, 28, 30–33]. Compared to the laparoscopic approach, the open approach was associated with a higher rate of SSI (RR 0.47; 95% CI 0.23 to 0.96, *P* = 0.04), with no between-study heterogeneity (*I*^2^ = 0, *P* = 0.78).

AL was evaluated in seven studies [24, 26, 28, 30–32], with no between-study heterogeneity (*I*^2^ = 0, *P* = 0.99). The overall effect test showed an RR of 0.82 (95% CI 0.37 to 1.84; *P* = 0.64).

The analysis of additional primary outcomes, such as postoperative pulmonary infection, postoperative wound dehiscence, and time to first flatus, was hindered by insufficient data for inferential analysis. Postoperative pulmonary infection was reported in six studies [24, 26–28, 31, 33]; however, inconsistencies in the definitions of this outcome across the studies were noted. Specifically, one study [26] defined it as a pulmonary complication, another [27] as pleural effusion, while the remaining four studies [24, 28, 31, 33] classified it as pulmonary infection. To mitigate bias, an analysis of these outcomes was not conducted. Furthermore, postoperative wound dehiscence was documented in only three studies [16, 26, 28], and time to first flatus was reported in four studies [16, 28, 31, 33]. The limited number of studies addressing these outcomes precluded further analysis.

### Subgroup and sensitivity analyses

Meta-regression analysis was conducted to explore factors that potentially influence heterogeneity, including the types of surgeries, tumor sites, and NOS scores. The findings indicated that the types of surgeries significantly contributed to the heterogeneity of operation time (*P* < 0.001). Subsequently, subgroup analysis was performed to further investigate significant heterogeneity based on the types of surgeries, and sensitivity analysis was conducted using the one-by-one exclusion method.

The study analyzed the operation time, intraoperative bleeding, and LOS based on the type of surgery performed (radical or not). Detailed information can be found in Table 3. The results showed that among patients undergoing radical surgery, there was no significant difference in the operation time between the two groups. This finding deviated from the overall results, and there was a lower heterogeneity between studies (*I*^2^ = 0). Although there was no significant difference in intraoperative blood loss between the two groups, there was substantial heterogeneity (*I*^2^ = 89%). Regarding LOS, the laparoscopic approach group exhibited a shorter hospital stay than the open approach group in the radical surgery subgroup. However, there was also significant heterogeneity among the studies (*I*^2^ = 89%).

**Table 3:** Subgroup analysis

Outcomes	Trials*	Participants	Analysis model	Results		Heterogeneity	
				Mean difference (95% CI)	*P* value	*I*^2^ (%)	*P* value
Operation time	10 [16, 25, 26, 28–33, 35]	875	Random	20.88 (2.31, 39.44)	0.03	90	< 0.0001
Types of surgery							
Radical	4 [26, 28, 31, 33]	448	Random	3.07 (− 4.82, 10.96)	0.45	0	0.40
Mixed	6 [16, 25, 29, 30, 32, 35]	427	Random	20.88 (2.31, 39.44)	0.22	29	0.22
Intraoperative blood loss	9 [16, 25, 28–32, 35]	781	Random	− 25.84 (− 52.16, 0.49)	0.05	73	0.00020
Types of surgery							
Radical	3 [28, 31, 33]	354	Random	− 9.96 (− 45.78, 25.85)	0.59	89	< 0.0001
Mixed	6 [16, 25, 29, 30, 32, 35]	781	Random	− 45.06 (− 69.56, − 20.81)	0.0003	0	0.72
LOS	9 [16, 25, 26, 28–33]	857	Random	− 3.29 (− 5.27, − 1.31)	0.001	89	< 0.00001
Types of surgery							
Radical	4 [26, 28, 31, 33]	448	Random	− 2.55 (− 4.79, − 0.31)	0.03	85	0.0002
Mixed	5 [16, 25, 29, 30, 32]	409	Random	− 3.99 (− 8.06, 0.09)	0.06	89	< 0.00001

*LOS* length of hospital stay

*Please refer to the manuscript for the cited references

Sensitivity analysis of the one-by-one exclusion method was conducted to examine the impact of greater heterogeneity in the population and radical subgroups on the results. Our findings reveal that excluding Kim [29] and Mat [24] did not significantly alter the overall heterogeneity of operation time. However, the overall results differed significantly (MD = 18.92, 95% CI − 2.18 to 40.02; *P* = 0.08), (MD = 18.69, 95% CI − 0.41 to 37.79; *P* = 0.06). Thus, it can be suggested that the overall results are not reliable, but the results of the radical subgroup remain stable.

Based on a meticulous analysis of the total and specific subgroups of intraoperative blood loss, our findings indicate that there is considerable heterogeneity and variability in the results, with only a small number of studies remaining unaffected. This leads us to conclude that the reliability of both the overall and specific subgroup results of intraoperative blood loss is questionable.

Furthermore, in order to conduct a sensitive analysis, we excluded the results of LOS for both the overall and specific subgroups individually. It is worth noting that none of these exclusions had a significant impact on the results, suggesting their stability.

## Discussion

Upon review of the literature [36], the use of a stent as BTS is designed to decompress the large bowel in acutely ill patients, thereby providing time for patient stabilization, diagnostic staging, bowel cleansing, and the transition from an emergency to an elective surgical intervention. The attenuation of intestinal edema, enhancement of the patient’s nutritional status, and bolstered immunity collectively facilitate the subsequent employment of minimally invasive surgical techniques once the intestinal obstruction is alleviated. Currently, there is a dearth of published randomized trials evaluating the surgical modalities for CRC resection in an emergency context. Furthermore, comprehensive meta-analytical reviews of SEMS placement remain absent. To our knowledge, this meta-analysis represents the inaugural effort to elucidate the correlation between surgical approaches and clinical outcomes, aiming to draw significant conclusions through meta-analytical methods.

For primary outcomes, this meta-analysis demonstrated that laparoscopic surgery significantly reduced the rates of overall POCs (RR 0.52; 95% CI 0.37 to 0.72, *P* < 0.0001). Additionally, there was no significant difference in 30-day mortality when comparing the open surgery group to the laparoscopic group (RR 1.09; 95% CI 0.26 to 4.48; *P* = 0.95). Hence, laparoscopic surgery post-stent insertion for OCRC patients is deemed safe and viable.

Concerning secondary outcomes, which encompassed perioperative metrics, postprocedural adverse events, and LN dissection, the laparoscopic group showed a reduction in LOS (MD − 3.29; 95% CI − 5.27 to 1.31; *P* = 0.001), intraoperative blood loss (MD − 25.84, 95% CI − 52.16 to 0.49; *P* = 0.05), and postoperative SSI (RR 0.47; 95% CI 0.23 to 0.96, *P* = 0.04) compared to the open surgery group. No significant differences were noted in LN dissection, POI, or AL between the two groups. Open surgery, however, required less operative time than the laparoscopic approach (MD 20.99, 95% CI 2.31 to 39.44; *P* = 0.03). High heterogeneity in the results for operative time, intraoperative blood loss, and LOS prompted a meta-regression, which identified the type of surgery as the source of this heterogeneity. Consequently, we conducted a subgroup analysis based on this finding for two primary reasons: (1) such analysis significantly reduces heterogeneity and (2) the established approach for curative surgery in colon cancer involves Colectomy with en bloc removal of regional lymph nodes [37]. The resection should encompass a segment of the colon, extending at least 5 cm on either side of the tumor, with the excision of a minimum of 12 lymph nodes [38]. Guided by established protocol [39], TME is a fundamental element of radical rectal cancer surgery. From a clinical perspective, radical surgery is a standardized procedure with considerable practical importance.

Upon stratifying the outcome of operation time and intraoperative blood loss according to surgical type, we found that despite overall results being statistically significant, no significant differences were observed in the radical subgroup. Researchers [40] suggest that the proficiency gain curve for self-taught senior surgeons performing laparoscopic colorectal surgery lies between 100 and 150 procedures. This learning curve might account for why the outcomes of operation time and intraoperative blood loss for the laparoscopic approach were not superior to those of the open approach in the radical subgroup. Other potential factors could include the improved general condition of patients following SEMS implantation, which may not align with the conditions of routine elective patients due to factors, such as intestinal edema and nutritional status, thereby complicating the actual laparoscopic operation. Regrettably, the included studies neither quantified the degree of remission nor conducted a systematic comparison with conventional elective patients. More convincing results might be obtained if future studies grade the degree of obstruction and establish corresponding subgroups for analysis. It is noteworthy that our studies' statistical results related to intraoperative blood loss are highly heterogeneous and the sensitivity analysis results are unreliable. The primary reasons include (1) a lack of standard descriptions for calculating intraoperative blood loss in the studies and (2) the presence of numerous confounding factors during calculation, such as varying amounts of abdominal lavage and intraoperative fluid loss among patients. As such, we recommend treating the result of intraoperative bleeding with caution.

Resection of rectal cancer is technically more challenging than colon cancer resection [41]. A study [42] highlighted that laparoscopic procedures, which often employ long, straight instruments, inherently face limitations during the surgical excision of rectal cancers situated deep within the pelvis. These limitations include a restricted range of motion, diminished dexterity, and uncontrolled traction by assistants. Another study [1] noted similar challenges with laparoscopic surgery for rectal tumors, specifically due to limited pelvic exposure and the inherent limitations of instrumentation in obese patients or male patients with a narrow pelvis. Regrettably, our subgroup analysis could not isolate obstructions caused by rectal cancers due to the paucity of studies focusing solely on obstructive rectal cancer.

Clinical practice guidelines from the AGA [12] stated that despite growing evidence supporting the use of SEMS in left-sided malignant obstructions, concerns remain regarding their application for right-sided or proximal colon tumors due to the technical complexities associated with SEMS insertion in these areas. Most studies have focused on LSOCC and rectal cancers [6], whereas few have reported on right-sided malignant obstructions. Emergency resection is generally considered safe for acute obstructing right-sided colon cancer. The findings of a recent meta-analysis [43] reveal that BTS for right-sided malignant large bowel obstruction (MLBO) yields more favorable short-term outcomes compared to left-sided MLBO. This implies that BTS leads to a decrease in postoperative complications and mortality for right-sided MLBO when compared to emergency resection. While current evidence supports the feasibility of BTS for right-sided obstructive colon cancer, limited studies assessing its safety and effectiveness impede the establishment of definitive conclusions. Unfortunately, our subgroup analysis was also unable to conduct a focused analysis on obstructions caused by right colon cancer due to the absence of independent studies.

Our findings indicate that the laparoscopic group did not exhibit superiority over the open group concerning POI, AL, and LN dissection. Abdominal surgery has been shown to activate muscularis macrophages and inflame the intestinal muscle layer, resulting in compromised contraction and motility [44, 45]^.^. The extent of tissue damage is influenced by the degree of intestinal manipulation and the duration of surgery, thereby affecting the severity of POI. In mice, laparoscopic surgery did not induce intestinal inflammation and POI as compared to standard intestinal manipulation [46, 47]. Open surgery, in contrast to minimally invasive surgery (MIS), has been shown to substantially increase the likelihood of POI, with OR ranging from 1.97 to 6.37 [48]. However, our research yielded a divergent result, indicating no statistically significant difference between the two groups.

Incomplete resolution of bowel wall edema is posited to contribute to the incidence of AL. AL continues to pose a significant risk to patients, particularly at high-risk sites, such as colorectal areas [49]. Limitations of minimal access intracorporeal anastomosis include the absence of direct tactile sensation, insufficient exposure, and a suboptimal cutting angle of the endo-linear stapler [50]. Recent meta-analyses [1, 51] comparing techniques for rectal cancer resection found no significant difference in anastomotic leak between the laparoscopic and open approaches. Conversely, a meta-analysis by Qu et al. [52] suggested that laparoscopy may be associated with a lower risk of AL than open surgery. Our research concurred with the former, finding no statistical difference between the two groups. One potential explanation is that laparoscopic surgery inflicts less tissue trauma and is correlated with more favorable immunological outcomes and a reduced inflammatory response, potentially enhancing anastomotic healing and decreasing leakage rates. However, laparoscopic surgeries, particularly in rectal cancers with a narrow pelvis, may tend to employ multiple staplers.

In open surgery, anastomotic tension can be readily evaluated before anastomosis, often leading to additional proximal resection beyond the oncological safety margin. Conversely, in laparoscopic surgery, the extent of proximal resection is constrained by the requirement for tension-free anastomosis when planning an anastomosis. Despite the potential challenges posed by the use of in-line instrumentation in the pelvis during laparoscopic surgery, the benefits of enhanced access and visualization in the mid to lower rectum should not be overlooked [53]. Several meta-analyses have compared techniques for rectal cancer resection and found no significant difference in the number of nodes retrieved between laparoscopic and open methods [1, 54]. An RCT [55] on stage II/III colon cancer reported no significant differences in the number of dissected LN between open surgery and laparoscopic surgery groups. Another study [51] showed fewer dissected LN in the laparoscopic group for advanced low rectal cancer compared to the open group. However, our results found no significant difference between the two groups.

The optimal time interval between stent implantation and subsequent surgery for patients with OCRC remains undefined [56]. This variable, which is within our control, influences surgical strategy and impacts tumor prognosis. Some research [7, 57] suggests a higher risk of AL when the interval between SEMS placement and surgery is brief. An extremely short time interval (< 7 days) appears associated with POCs due to insufficient intestinal decompression and systemic recovery [58]. A longer gap between SEMS insertion and surgery allows patient condition stabilization, accurate disease staging, effective bowel decompression, and resolution of bowel obstruction symptoms, possibly leading to improved surgical outcomes in a BTS setting [59]. However, a longer duration of stent placement could theoretically cause silent and micro-perforations, leading to tumor cell dissemination through the bloodstream and poorer oncological outcomes [35]. One retrospective cohort study [60] suggested that longer intervals (> 14 days) between SEMS placement and surgery did not affect surgical difficulty but improved the rate of primary anastomosis and reduced the rate of stoma creation and postoperative complications. Ho et al. [61] reported an increased risk of surgery if the interval exceeded two weeks due to significant peritumor inflammation and fibrotic adhesions caused by the SEMS. Some studies [62, 63] have also found no correlation between varying time intervals and postoperative complications. A recent meta-analysis [43] reveals that an interval of 20 days or more between the placement of SEMS and surgery significantly lowers the occurrence of postoperative complications. An extensive national cohort study [64] of patients diagnosed with LSOCC indicates that achieving an optimal equilibrium between SEMS-related complications and patient recovery, under optimized surgical conditions, is likely attained by scheduling the resection approximately 2 weeks after successful SEMS placement. The ESGE guideline [6] recommends the use of uncovered SEMS in the curative setting and a two-week interval between colonic SEMS placement and resection. The guideline also notes the lack of high-quality literature to grade obstruction severity. The timing of surgery following colonic stenting must consider the balance between stent-related adverse events (reduced by a short interval) and surgical outcomes (improved by a longer delay). Accurate grading of obstruction before and after SEMS placement may guide the length of this interval. Exploring the specific bridging interval for obstructive malignant tumors at different sites of the large bowel (left side, right side, and rectum) may necessitate further investigation.

Chemotherapy reduces the risk of tumor ingrowth compared to SEMS use alone; however, it is also associated with long-term complications, like perforation and stent migration. The decision to combine neoadjuvant chemotherapy during the interval as a treatment is poorly studied [58]. A meta-analysis [65] indicated that neoadjuvant chemotherapy (NAC) was not associated with improved survival outcomes, despite its safety and feasibility in perioperative management. There is also a potential risk of emergency surgery during NAC, particularly in patients with tumor progression or adverse conditions due to chemotherapy. Further optimization of clinical staging is crucial to accurately identify patients who may benefit from neoadjuvant therapy and avoid overtreatment of low-risk patients.

This study acknowledges several limitations that warrant consideration: (1) Conducting RCTs for patients with OCRC is challenging. Consequently, the studies included in this article are either retrospective or prospective cohort studies. As a result of these research design constraints, there may be a high risk of selection, implementation, and measurement biases. Patients with complete obstruction may not attempt stent placement. Furthermore, during the pre-operative evaluation following stent implantation, if a patient is deemed at high surgical risk, the surgical plan may be modified to avoid elective surgery. Currently, there is no objective standard for determining the surgical approach, and the choice between open surgery and laparoscopic surgery is largely dependent on the surgeon's preference, potentially causing selection bias. Additionally, the varying quality of studies and insufficient sample sizes in some may undermine the credibility of the results; (2) there is heterogeneity in the population definition and some outcome indicators included in the study. The limited number of studies precludes further subgroup analysis based on factors, such as tumor location, specific operation mode, interval between stent insertion and operation, stent type, obstruction degree, and relief degree of obstruction post-stent implantation. These areas represent directions for future research; (3) the clinicians involved in the SEMS program differ. Stenting is performed by endoscopists, interventional radiologists, and colorectal surgeons. Owing to the lack of detailed information on endoscopic physician composition in most studies, meaningful comparisons between different clinician groups are unfeasible; (4) most of the studies included do not specify clear follow-up times or have short follow-up periods, limiting the evaluation of equivalent later indicators, and the long-term effects remain uncertain.

## Conclusion

In summary, for patients diagnosed with OCRC, laparoscopic surgery marginally outperforms open surgery by diminishing postoperative risks and abbreviating hospital stays. However, to substantiate these findings, research of superior quality is imperative. Furthermore, it is essential to ascertain the ideal interval between stent implantation and surgery, as well as to refine the clinical staging of patients for a more precise evaluation of neoadjuvant therapy risks.

### Supplementary Information

Below is the link to the electronic supplementary material.

Supplementary file1 (DOCX 14 KB)
