# Supplementary material for: Laparoscopic versus open surgery in obstructive colorectal cancer patients following stents placement: a comprehensive meta-analysis of cohort studies
Source: Surg Endosc. 2024 Mar 5;38(4):1740–57. doi: 10.1007/s00464-024-10710-4 (PMC10978680; doi:10.1007/s00464-024-10710-4)
Supplement: Supplementary file 1 — Supplementary file1 (DOCX 14 KB) [file 464_2024_10710_MOESM1_ESM.docx]

#1 "colorectal neoplasms" [MeSH Terms]

#2 colorect* OR colon* OR rect* OR anal* OR anus* OR intestin* OR bowel*

#3 carcinom* OR neoplas* OR adenocarcinom* OR cancer* OR tumor* OR tumour* OR sarcom* OR adenom* OR malignan* OR polyp*

#4 #2 and #3

#5 #1 or #4

#6 Intestinal Obstruction [MeSH Terms]

#7 bowel* OR intestin* OR gastrointestin* OR gastro-intestin* OR colon* OR colorect* OR retrosigmoid* OR acute

#8 obstruct* OR occlu* OR fail* OR block* OR adhes* OR stenoses OR stenosis OR stricture

#9 #7 and #8

#10 #6 or #9

#11 Stents [MeSH Terms]

#12 (stent* or SEMS or SEMT* or "Bridge to surgery")

#13 #11 or #12

#14 laparoscopy [MeSH Terms]

#15 (laparoscop* or laparotom*)

#16 #14 or #15

#17 (((((Minimal Access Surgical [Title/Abstract]) OR (Minimal Surgical [Title/Abstract])) OR (Minimally Invasive [Title/Abstract])) OR (Surgical Procedures, Minimal [Title/Abstract])) OR (Surgical Procedure, Minimal [Title/Abstract])) OR (minimally invasive surgery [MeSH Terms])

#18 (((((Elective surgeries [Title/Abstract]) OR (Elective surgery [Title/Abstract])) OR (Surgical Procedure, Elective [Title/Abstract])) OR (Elective Surgical [Title/Abstract])) OR (Surgical Procedures, Elective [Title/Abstract])) OR (Elective Surgical Procedures [MeSH Terms])

#19 #16 OR #17 OR #18

#20 #5 AND #10 AND #13 AND #19

#21 (animals [MeSH Terms]) NOT (humans [MeSH Terms])

#22 #20 NOT #21
